# Synthetic Seismic Accelerogram Generation via Wavelet- Decomposed Conditional Generative Adversarial Networks

**DOI:** 10.3390/s26123725

**Published:** 2026-06-11

**Authors:** Antonio Rocca, Luigi Laura, Marco Parrillo

**Affiliations:** Computer Engineering, Faculty of Engineering, Università Telematica Internazionale UNINETTUNO, 00186 Rome, Italy; roccaab@gmail.com

**Keywords:** synthetic accelerograms, generative adversarial networks, discrete wavelet transform, seismic signal synthesis, conditional GAN, deep learning, earthquake engineering

## Abstract

The generation of synthetic seismic accelerograms is a critical problem in earthquake engineering, where the scarcity of strong-motion records, particularly for high-magnitude and near-fault scenarios, limits the reliability of structural analyses and probabilistic seismic hazard assessments. This paper presents a proof-of-concept wavelet-decomposed conditional Generative Adversarial Network (WD-cGAN) for the synthesis of seismic accelerograms that reproduce the physical and statistical properties of real ground-motion records. Unlike prior GAN-based approaches that rely on Fourier-domain decomposition, the proposed architecture decomposes each training signal into *N* wavelet sub-bands (experimentally N=7, six detail sub-bands D1–D6 and one approximation sub-band A6) using the Daubechies-4 (db4) discrete wavelet transform (DWT), assigning each sub-band to a dedicated discriminator. A novel energy-based weighting scheme $\alpha_{i}$ modulates the relative contribution of each discriminator to the total generator loss, ensuring that physically dominant, low-frequency bands, which carry the bulk of seismic energy, receive proportionally higher training emphasis. Seismic moment magnitude $M_{w}$ serves as the primary conditioning variable, enabling targeted synthesis for specific hazard scenarios. The model is implemented in Python v3.9 using PyTorch v.2.10 and trained on accelerograms drawn from the Italian INGV/ITACA v4.0 archive. Preliminary evaluation on 500 synthetic accelerograms across five magnitude classes provides evidence that the proposed wavelet-domain multi-discriminator scheme reproduces the essential spectral shape and non-stationary temporal structure of real ground-motion records within the considered magnitude range; full quantitative validation on a larger and more diverse corpus, rigorous comparison with competing methods, and extended multi-parameter conditioning are identified as the principal avenues for future work.

## 1. Introduction

Seismic accelerograms, time-series records of ground acceleration during earthquake events, constitute the primary input for time-history structural analyses, seismic risk assessments, and performance-based earthquake engineering methodologies. Three categories of accelerograms are employed in practice: natural records from instrumented events; artificial signals generated by stochastic algorithms to match target response spectra; and physics-based synthetic signals produced by numerical simulation of source rupture and wave propagation [1]. Each category carries well-known limitations. Natural records are scarce for high-magnitude, near-fault, and site-specific scenarios, resulting in an insufficient number of statistically representative samples for robust hazard quantification. Artificial signals, although spectrally compatible, often lack physically realistic phase characteristics and non-stationary amplitude envelopes. Physics-based simulations are computationally expensive and require detailed knowledge of the regional crustal model, which is seldom available with adequate resolution.

Early probabilistic deep generative models include variational autoencoders (VAEs), originally introduced by Kingma and Welling [2], and generative adversarial networks (GANs), proposed by Goodfellow et al. [3]. Among these approaches, GANs offer an attractive alternative for seismic signal generation. Once trained on an existing catalog, a GAN can produce statistically consistent new samples in negligible computational time. In its conditional formulation [4], the generation process can be guided by prescribed physical parameters, such as magnitude, hypocentral distance, or site class. This capability has direct implications for seismic data augmentation in regions with sparse instrumentation, for Monte Carlo-based fragility analyses, and for the development of ground-motion models in data-limited environments.

This capability has direct implications for seismic data augmentation in regions with sparse instrumentation, for Monte Carlo-based fragility analyses, and for the development of ground-motion models in data-limited environments. The broader applicability of GAN-based synthesis across scientific domains further motivates this line of investigation.

The principal challenge in applying GANs to seismic time series lies in the distinctive physical structure of the signals. Accelerograms are strongly non-stationary: they exhibit impulsive high-frequency arrivals associated with P- and S-waves, a sustained coda of intermediate frequency, and a long-period, low-frequency tail dominated by surface waves. These features coexist in time and are spread across several decades of frequency, making any global spectral representation fundamentally inadequate.

Existing GAN-based approaches to seismic signal generation predominantly employ the Fourier transform for pre-processing, either through direct frequency-domain conditioning or Fourier-based loss functions [5]. The Fourier transform, however, provides only a global spectral representation: temporal information is lost, and non-stationary signal structure cannot be encoded. The discrete wavelet transform (DWT) [6,7] provides, by contrast, a multi-resolution time-frequency decomposition that preserves the temporal localization of spectral features. This property makes the DWT particularly well-suited to the characterization of seismic ground motion, as recognized in the seismological literature [5,8].

This paper makes the following contributions:

1. A wavelet-decomposed conditional GAN architecture (WD-cGAN) in which each DWT sub-band of the training accelerogram is evaluated by a dedicated discriminator, enabling specialized, frequency-localized adversarial training.
2. An energy-based discriminator weighting scheme that adaptively assigns scalar weights $\alpha_{i}$ proportional to the energy content of each wavelet component, ensuring that the generator gradient is dominated by physically significant frequency bands rather than by high-frequency noise.
3. A complete experimental evaluation on records from the Italian INGV/ITACA v4.0 archive, comprising qualitative and quantitative assessment of synthetic signal fidelity across five magnitude classes and covering spectral, temporal, and distributional metrics; all described experiments have been executed and are fully reported in Section 6.
4. A structured discussion of limitations and a concrete roadmap for future work, including architecture search, physics-informed constraints, and extended conditioning.

The remainder of the paper is organized as follows. Section 2 reviews related work. Section 3 provides necessary background on seismic time series, wavelet analysis, and GANs. Section 4 describes the proposed WD-cGAN architecture. Section 5 details the dataset and pre-processing pipeline. Section 6 presents experimental results. Section 7 and Section 8 discuss findings and future directions, respectively. Section 9 concludes the paper.

## 2. Related Work

### 2.1. GAN-Based Seismic Signal Generation

The application of deep generative models to seismic data has attracted growing interest over the past decade. Goodfellow et al. [3] introduced the GAN framework in the context of natural image synthesis; its extension to one-dimensional physical time series required architectural adaptations, including 1-D convolutional layers and temporal batch normalization. Within the seismological domain, early work employed variational autoencoders and simple fully connected architectures to synthesize ground-motion waveforms, with limited success in reproducing non-stationary characteristics. Classical stochastic simulation methods, such as the non-stationary Sabetta–Pugliese procedure [9], generate spectrum-compatible accelerograms by modulating a filtered noise process with an empirical amplitude envelope; they remain widely used in Italian seismic engineering practice and serve as a natural baseline for comparison with data-driven approaches.

More recent studies have adopted convolutional GANs and introduced frequency-domain conditioning. Esfahani et al. [10] proposed TFCGAN, a conditional GAN operating in the time-frequency domain via short-time Fourier transform (STFT) conditioning, demonstrating non-stationary ground-motion synthesis with explicit spectral supervision. Florez et al. [11] trained a data-driven broadband synthesis model on a large Southern California catalog, achieving high spectral fidelity over a wide magnitude and distance range. Matinfar et al. [12] applied a deep convolutional GAN to generate spectrum-compatible accelerograms from a limited number of records, a data-availability setting comparable to the present study. While these approaches represent meaningful advances, they share a common reliance on Fourier or STFT representations, whose fixed time-frequency resolution is a fundamental limitation for signals whose spectral content evolves on multiple timescales simultaneously, as is the case for seismic accelerograms [5].

### 2.2. Wavelet Methods in Seismology

Wavelet analysis has an established tradition in seismological signal processing. Kumar and Foufoula-Georgiou [5] provided a comprehensive review of wavelet methods for geophysical applications, demonstrating their superiority over Fourier techniques for the analysis of non-stationary geophysical signals. Baker [8] applied the continuous wavelet transform to the quantitative classification of near-fault ground motions, confirming that wavelet-domain features are physically interpretable and correlate with observed structural damage patterns. The DWT, in particular, has been used for seismic denoising, P-wave onset detection, and source characterization [6,7].

### 2.3. Multi-Discriminator and Multi-Scale GAN Architectures

The use of multiple discriminators operating at different scales was pioneered in the image-synthesis literature, where multi-scale discriminators were shown to stabilize training and improve detail reproduction at multiple resolutions. In the time-series domain, analogous multi-scale designs have been proposed for audio waveform synthesis and electrocardiogram generation. The present work adapts this paradigm to seismic signals, with the key distinction that the decomposition is performed in the wavelet domain rather than by simple downsampling, and that discriminator weights are derived from physical energy arguments rather than from uniform or learned aggregation.

### 2.4. Long Short-Term Memory and Temporal Convolutional Networks

Recurrent architectures, particularly Long Short-Term Memory networks (LSTMs) [13], have been applied to seismic phase detection and event classification; their capacity to model long-range temporal dependencies makes them natural candidates for discriminating low-frequency seismic components. Temporal convolutional networks (TCNs) [14] have been proposed as computationally efficient alternatives with comparable or superior performance on sequence modeling benchmarks. The present study employs convolutional discriminators throughout, with LSTM-based and TCN-based discriminators identified as a priority for future development.

## 3. Background

### 3.1. Seismic Time Series

An accelerogram is a discrete-time series sampled at regular intervals. The INGV/ITACA dataset used in this work is sampled at $f_{s}=200\mathrm{Hz}$ (sampling interval Δt=0.005s). Each record provides the three-component ground acceleration (e,n,z) corresponding to the East, North, and Vertical directions. Components are normalized as:

(1) $$\left[\hat{e},\;\hat{n},\;\hat{z}\right]=\left[\frac{e-\bar{e}}{\sigma_{e}},\frac{n-\bar{n}}{\sigma_{n}},\frac{z-\bar{z}}{\sigma_{z}}\right],$$

where $\bar{\left(\;\cdot \;\right)}$ and $\sigma_{\left(\;\cdot \;\right)}$ denote the sample mean and standard deviation of each component, respectively.

Seismic signals exhibit four defining characteristics that make generative modeling particularly challenging:

1. **Non-stationarity**: amplitude, frequency content, and statistical moments evolve continuously throughout the event.
2. **Piecewise impulsivity**: rapid, high-amplitude transients associated with P-wave and S-wave arrivals.
3. **Inherent stochasticity**: aleatory variability arising from source rupture heterogeneity and path-specific wave propagation.
4. **Internal irregularity**: complex scattering and interference of seismic waves interacting with heterogeneous geological structures.

It is important to note that accelerograms in the utilized database often begin with several seconds of non-significant signal, inducing imbalance in the neural networks. Removing silent portions would likely improve performance but conflicts with the fixed-length input requirement of GANs. In the current implementation, a fixed-length symmetric window (−t,+t) centered on the sample of peak ground acceleration (PGA) is extracted from each record, with t=20 s. This strategy maximizes the information content provided to the model by focusing on the most physically informative portion of the record, reduces the influence of non-informative segments present in the raw recordings, and preserves the fixed input length required by the GAN architecture. Alternative centering criteria, such as alignment on the S-wave arrival estimated via STA/LTA detection, may further improve training stability and are identified for future investigation.

### 3.2. Discrete Wavelet Transform

The discrete wavelet transform [6,7] decomposes a signal into a set of approximation and detail coefficients at multiple resolution levels. For a signal x[n] decomposed to level *L*, the perfect reconstruction relation is

(2) $$x\left[n\right]=A_{L}\left[n\right]+\sum_{l=1}^{L}D_{l}\left[n\right],$$

where $A_{L}$ denotes the level-*L* approximation (low-frequency residual) and $D_{l}$ denotes the detail coefficients at resolution level *l*.

The lower cutoff frequency associated with detail coefficient $D_{i}$ is given by Equation (3):

(3) $$F_{l}=\frac{f_{s}}{2^{l+1}},$$

for a sampling frequency of 200 Hz, the resulting wavelet sub-bands are: $D_{1}$ (50–100 Hz), $D_{2}$ (25–50 Hz), $D_{3}$ (12.5–25 Hz), $D_{4}$ (6.25–12.5 Hz), $D_{5}$ (3.125-6.25 Hz), $D_{6}$ (1.56–3.125 Hz), while $A_{6}$ contains frequency components below approximately 1.56 Hz. These frequency intervals represent the theoretical dyadic decomposition associated with the DWT.

**Figure 1:**
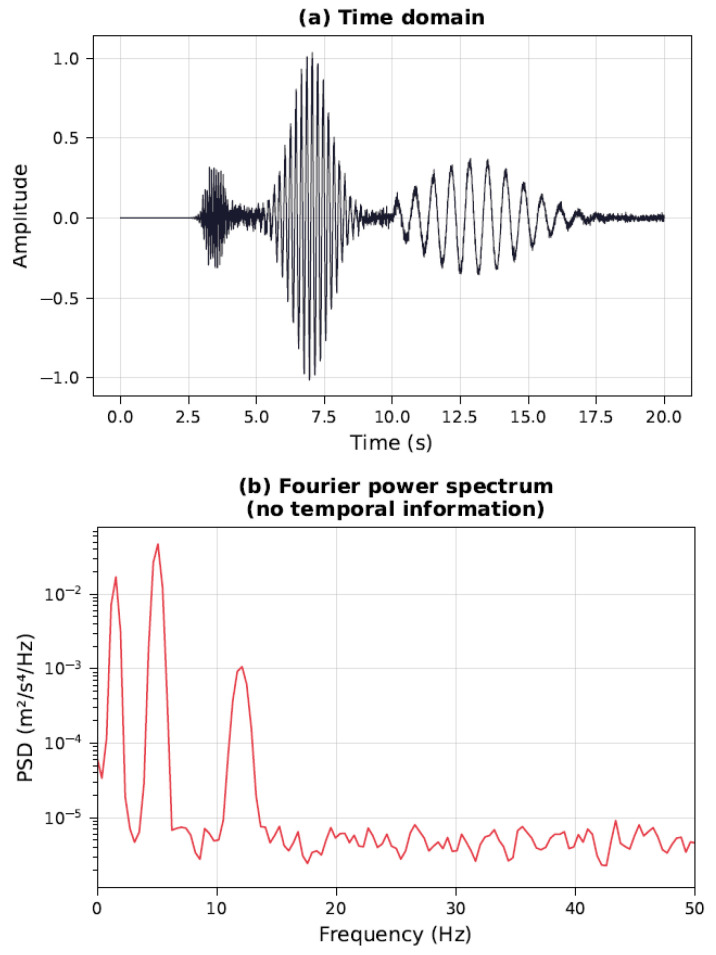
Comparison of two representations of a synthetic seismic accelerogram. (**a**) Time-domain waveform, which shows the temporal evolution of the signal but does not explicitly describe its frequency content. (**b**) Fourier power spectrum, which shows the global frequency content but removes any information on the temporal localization of the spectral components. The complementary limitations of these two representations motivate the wavelet-based sub-band decomposition adopted in the proposed WD–cGAN architecture.

The Daubechies-4 (db4) wavelet was selected for its compact support, regularity, and its established application in seismological analysis [6]. Compared to the short-time Fourier transform, the DWT provides adaptive time-frequency resolution: high temporal resolution at high frequencies and high spectral resolution at low frequencies, a property that is essential for capturing both the impulsive onset and the slow surface-wave coda of seismic records [5,8]. This contrast is illustrated in Figure 1, which compares the time-domain waveform and the Fourier power spectrum of a representative synthetic accelerogram; the complementary limitations of both representations motivate the wavelet-based sub-band decomposition adopted in the proposed WD-cGAN.

### 3.3. Generative Adversarial Networks

A standard GAN [3,15] trains a generator *G* and a discriminator *D* via the minimax objective

(4) $$\min_{G}\;\max_{D}\;V\left(D,G\right)=E_{x∼p_{\mathrm{data}}}\;\left[\log D(x)\right]+\;E_{z∼p_{z}}\;\left[\log\;\left(1-D(G(z))\right)\right].$$

Training converges, in theory, to a Nash equilibrium at which *G* produces samples indistinguishable from real data and *D* outputs 1/2 for all inputs.

A conditional GAN (cGAN) [4] augments both the generator and the discriminator with a conditioning variable *y*, yielding the objective

(5) $$\min_{G}\;\max_{D}\;V\left(D,G\right)=E_{x∼p_{\mathrm{data}}}\;\left[\log D(x|y)\right]+\;E_{z∼p_{z}}\;\left[\log\;\left(1-D(G(z|y)|y)\right)\right].$$

This formulation enables the model to generate samples consistent with a specific physical class or satisfying prescribed quantitative constraints.

## 4. Proposed Architecture

### 4.1. Overview

**Figure 2:**
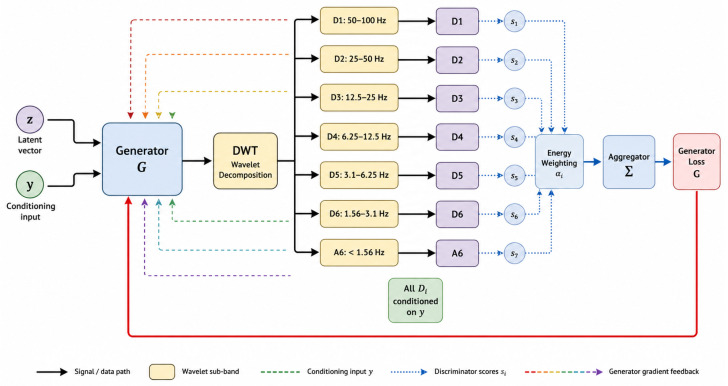
Overview of the wavelet-decomposed conditional GAN (WD-cGAN). The generator *G* produces a full-length accelerogram from a latent vector *z* and conditioning vector *y*. The DWT block decomposes the output into N=7 sub-bands; each sub-band is evaluated by a dedicated discriminator $D_{i}$ weighted by $\alpha_{i}$. Colored arrows indicate gradient feedback paths; the bold red arrow represents the aggregated generator loss signal.

The proposed WD-cGAN architecture consists of three principal components, illustrated schematically in Figure 2:

- A single generator *G* that maps a Gaussian latent vector $z\in R^{400}$, concatenated with a conditioning vector *y* encoding seismic parameters, to a full-length synthetic accelerogram $\hat{x}$.
- N=7 parallel discriminators $D_{1},\ldots ,D_{6},A_{6}$, each trained exclusively on one DWT sub-band of the signal, enabling frequency-localized adversarial evaluation.
- An energy-based weighting module that computes a scalar $\alpha_{i}$ for each discriminator from the energy content of the corresponding wavelet component, prioritizing physically dominant frequency bands during generator training.

### 4.2. Multi-Discriminator Objective

With *N* parallel discriminators, the generator objective is

(6) $$\max_{G}\;\sum_{i=1}^{N}\alpha_{i}\;E_{z∼p_{z}}\;\left[\log D_{i}\;\left(G(z)\right)\right],$$

and each discriminator independently maximizes

(7) $$\max_{D_{i}}\;E_{x∼p_{\mathrm{data}}}\;\left[\log D_{i}\left(x_{i}\right)\right]+\;E_{z∼p_{z}}\;\left[\log\;\left(1-D_{i}\left({\hat{x}}_{i}\right)\right)\right],$$

where $x_{i}$ and ${\hat{x}}_{i}$ denote the *i*-th wavelet sub-band of the real and synthetic signals, respectively. The weights $\alpha_{i}$ satisfy $\sum_{i=1}^{N}\alpha_{i}=1$ and are derived from the energy content of each sub-band, as described in Section 4.3.

### 4.3. Energy-Based Discriminator Weights

The energy of the *i*-th discrete wavelet component is defined as

(8) $$P_{i}=\sum_{n}{\left|x_{i}\left[n\right]\right|}^{2}.$$

The normalized weight assigned to discriminator $D_{i}$ is then

(9) $$\alpha_{i}=\frac{P_{i}}{\sum_{j=1}^{N}P_{j}}.$$

Because db4 is an orthogonal wavelet basis, the Parseval identity holds exactly:

(10) $${∥x∥}^{2}={∥A_{L}∥}^{2}+\sum_{l=1}^{L}{∥D_{l}∥}^{2}.$$

Equation (10) guarantees that the total signal energy is partitioned exactly across sub-bands, regardless of the number of coefficients in each band. A shorter sub-band with higher-amplitude coefficients, therefore, correctly dominates the energy sum, and no length-based normalization is required. The weights $\alpha_{i}$ are computed on the raw (un-normalized) wavelet coefficients immediately after decomposition (Step 2 of the pre-processing pipeline, Section 5.2), before the sub-band amplitude normalization applied for discriminator input in Step 3.

Components with higher energy receive proportionally higher weight, reducing the gradient contribution of high-frequency sub-bands that are typically dominated by anthropogenic noise and instrument artifacts. The physically motivated assignment is summarized as follows: sub-bands $D_{1}$ and $D_{2}$ (frequencies 25 Hz to 100 Hz) carry predominantly electrical and anthropogenic noise and receive low weight; sub-bands $D_{3}$ and $D_{4}$ (frequencies 6 Hz to 25 Hz) contain the principal P-wave and S-wave energy and receive moderate weight; sub-bands $D_{5}$ and $A_{6}$ (frequencies below 6 Hz) carry surface waves and the dominant seismic energy and receive the highest weight.

A representative accelerogram from the INGV/ITACA database was analyzed to assess the distribution of signal energy across the wavelet components used in the proposed WD–cGAN architecture. The signal was decomposed by means of a six-level db4 discrete wavelet transform, and the normalized energy weights $\alpha_{i}$ were computed for each sub-band. The analysis reveals a highly non-uniform energy distribution, with most of the energy concentrated in the lower-frequency components.

For the considered record, the high-frequency detail bands $D_{1}$, $D_{2}$, and $D_{3}$ contribute jointly less than 2% of the total signal energy. In contrast, the $D_{5}$ and $A_{6}$ components account together for approximately 80% of the total energy. This result supports the use of an energy-based weighting scheme in the adversarial loss, since the discriminators associated with energetically dominant sub-bands should exert a stronger influence on the generator update than those associated with nearly negligible components.

Accordingly, the proposed weighting strategy scales the contribution of each discriminator according to the relative energy content of its corresponding wavelet sub-band. This allows the generator loss to reflect the physical structure of the accelerogram more faithfully, emphasizing the frequency components that dominate the seismic response while maintaining multi-band adversarial supervision over the entire decomposed signal.

### 4.4. Conditioning on Seismic Parameters

The conditioning vector *y* is built from accelerogram metadata. Currently only moment magnitude $M_{w}$ is used; other parameters in Table 1 are reserved for future work. The embedded feature vector is concatenated with latent noise *z* before entering *G* and is also supplied to each $D_{i}$ with the corresponding wavelet sub-band, making generation and discrimination condition-aware.

Note: the z-score normalization in Equation (1) removes absolute amplitude information; the normalized signal is dimensionless and retains only temporal and spectral structure. Reconstructing physical accelerations ($\mathrm{cm}/s^{2}$), therefore, requires a target PGA, which depends on $M_{w}$, hypocentral distance *R*, and local site amplification ($V_{s30}$). Since these are not encoded in *y* in the present implementation, outputs are produced in normalized form and must be scaled to a PGA from a suitable GMPE for the region (e.g., Lanzano et al. [16]). The extended conditioning roadmap in Section 8 addresses this limitation.

### 4.5. Neural Network Architecture

Generator G.

**Figure 3:**
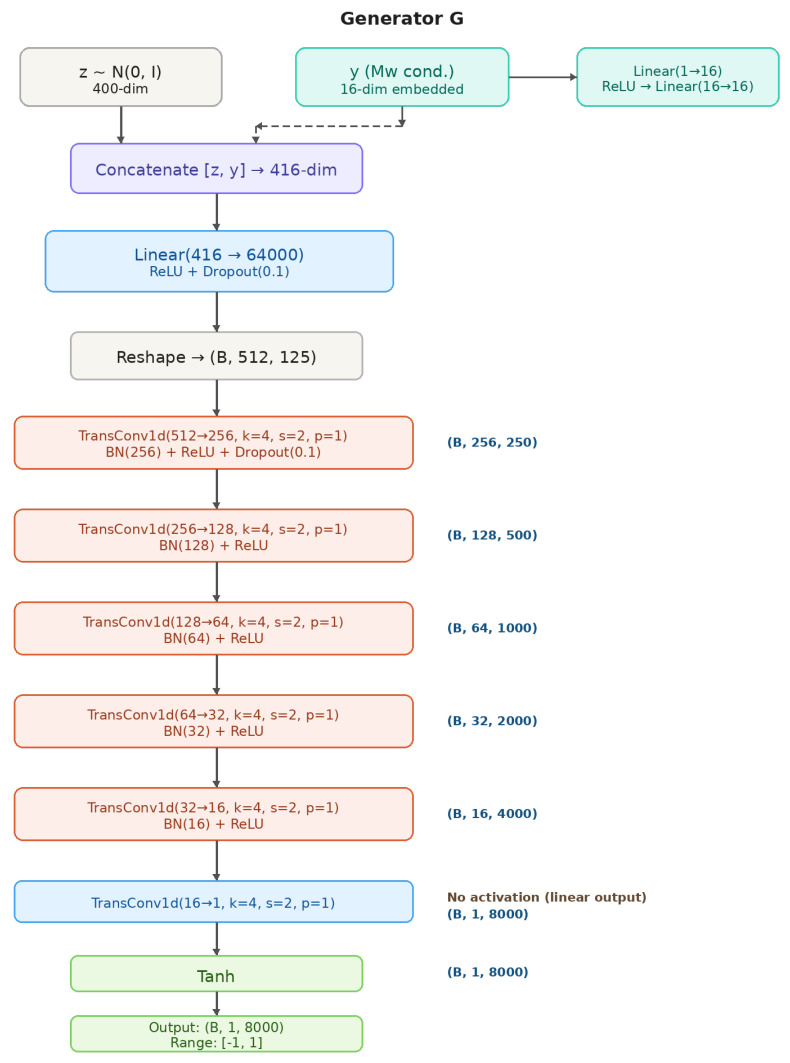
Generator architecture. The latent vector $z\in R^{400}$ is concatenated with the conditioning embedding $y\in R^{16}$ (derived from $M_{w}$ via a two-layer MLP) and projected to a 512×125 tensor through a fully connected layer with dropout. Six successive 1-D transposed convolutional blocks with batch normalization and ReLU activations progressively upsample the representation by a factor of 2 at each stage, producing a single-channel output of 8000 samples (40 s at $f_{s}=200$ Hz). No output activation is applied, allowing the generator to reproduce the full dynamic range of z-score normalized accelerograms.

The generator employs a stack of 1-D transposed convolutional layers with batch normalization and ReLU activations, progressively upsampling the concatenated latent and conditioning vector into a time series of the target length. The generator *G* follows a one-dimensional transposed-convolutional architecture that maps the latent vector z and the conditioning vector y to a full-length synthetic accelerogram. The full layer-by-layer specification is given in Table 2 and the architecture is illustrated in Figure 3.

Generator output activation.

The final Tanh activation layer was initially tested to constrain the generator output to the interval [−1,+1]. Although this bounded activation improves numerical stability, it was found to suppress high-amplitude transients in z-score normalized accelerograms. Therefore, the Tanh layer was removed to allow the generator to reproduce acceleration peaks more consistently with real seismic records.

Discriminators.

**Figure 4:**
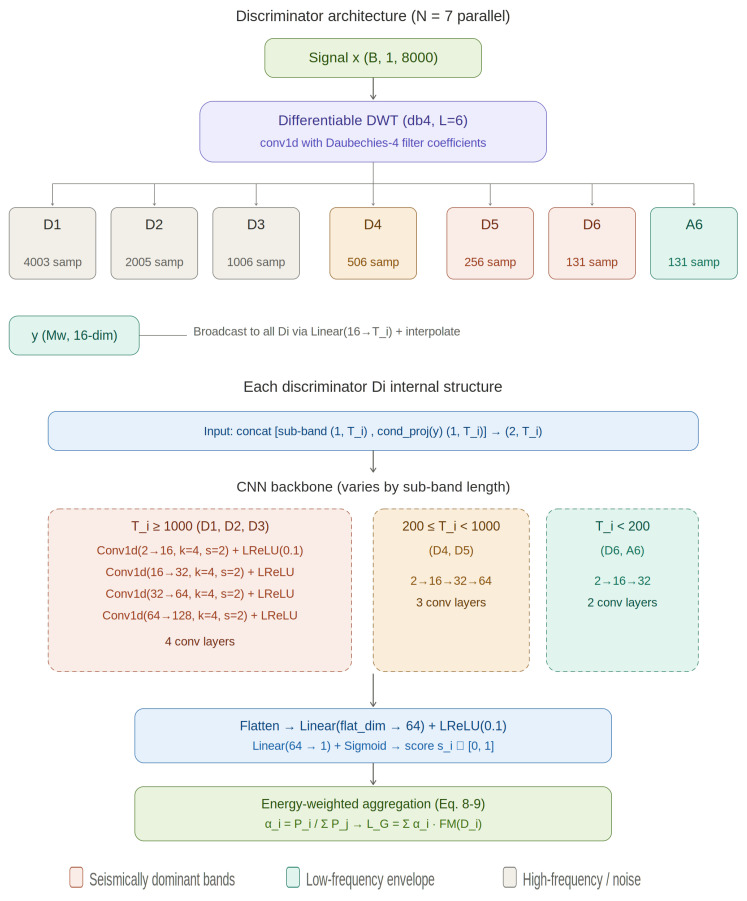
Discriminator architecture. The generated or real signal is decomposed into N=7 sub-bands by a differentiable DWT block (db4, L=6) implemented as conv1d operations. Each sub-band is evaluated by a dedicated discriminator $D_{i}$ that receives the wavelet coefficients concatenated with the conditioning vector y, linearly projected and interpolated to match the sub-band length $T_{i}$. The CNN backbone depth adapts to the input length: 4 layers for $T_{i}\geq 1000$ (D1–D3), 3 layers for $200\leq T_{i}<1000$ (D4–D5), and 2 layers for $T_{i}<200$ (D6, A6). Each discriminator outputs a scalar score $s_{i}\in \left[0,1\right]$ via a sigmoid head. Individual losses are aggregated using energy-based weights $\alpha_{i}$, ensuring that physically dominant sub-bands contribute proportionally.

Each discriminator $D_{i}$ receives a single wavelet sub-band as a 1-D signal of length $T_{i}$ determined by the decomposition level. The conditioning vector $y\in R^{16}$ is projected to length $T_{i}$ via a Linear(16, $T_{i}$) layer and concatenated with the sub-band as a second channel, giving a 2-channel input of shape $\left(2,\;T_{i}\right)$. The convolutional backbone depth adapts to $T_{i}$ as detailed in Table 3 and illustrated in Figure 4; the output is a scalar score s∈[0,1] via a fixed two-layer fully connected head with sigmoid activation. Recurrent architectures (LSTM; TCN) for the low-frequency discriminators $D_{4}$, $D_{5}$, and $A_{6}$ are planned for future development to better capture long-range temporal dependencies.

Conditioning mechanism.

Both *G* and each $D_{i}$ receive the feature vector *y*, rendering the architecture a multi-discriminator cGAN. Crucially, the multi-scale evaluation is not a substitute for conditioning; rather, each discriminator specializes in one frequency band and independently verifies consistency with the conditioning variable *y*, providing the generator with frequency-localized, condition-aware gradient feedback.

## 5. Data and Pre-Processing

### 5.1. Dataset

Training data were obtained from the Italian Accelerometric Archive (ITACA v4.0) [17], managed by the Istituto Nazionale di Geofisica e Vulcanologia (INGV). Records are provided in ASCII format and sampled at $f_{s}=200\mathrm{Hz}$. The selection criterion required $M_{w}\geq 4.0$ to ensure an adequate signal-to-noise ratio, yielding accelerograms from four seismic stations covering multiple Italian seismic sequences. Components HGE (East), HGN (North), and HGZ (Vertical) were retained. Automated data retrieval was implemented via the ITACA REST API.

The most significant training was performed using accelerograms from seismic events with $M_{w}\in \left[5.0,5.5\right)$, yielding 1879 accelerograms after quality filtering. The corpus was partitioned into training (70%), validation (20%), and test (10%) subsets using a stratified split by magnitude class, yielding 1315, 376, and 188 records, respectively.

The dataset is acknowledged to be small relative to those used in the broadband synthesis studies of Florez et al. [11] and Matinfar et al. [12]; this represents the primary limitation of the current work and is addressed in Section 8.

### 5.2. Signal Pre-Processing Pipeline

**Figure 5:**
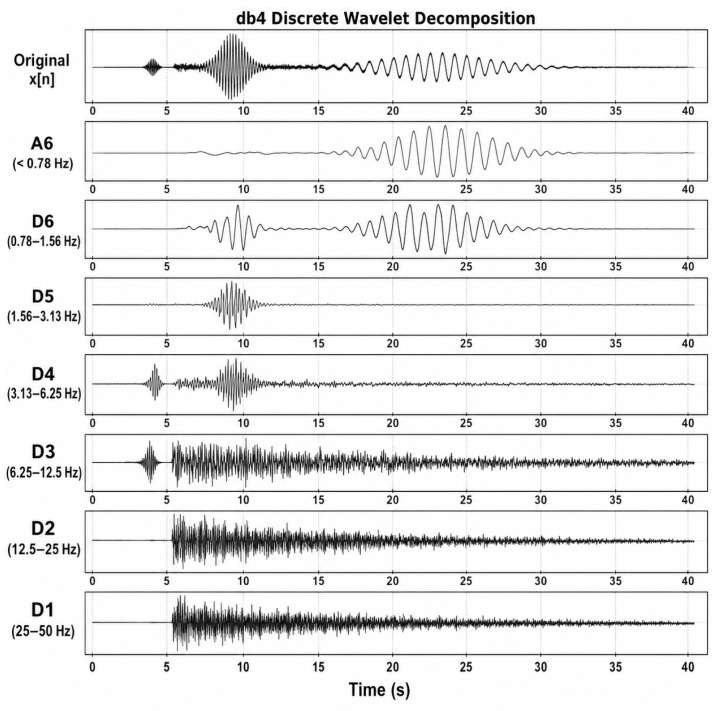
db4 discrete wavelet decomposition of a synthetic seismic accelerogram to level L=6. The top panel shows the original signal x[n]; subsequent panels show the approximation $A_{6}$ and detail sub-bands $D_{5}$ through $D_{1}$ in order of increasing frequency. Note the adaptive time-frequency resolution: $A_{6}$ captures the slow surface-wave envelope, while $D_{1}$ resolves the impulsive P-wave onset. All sub-bands are plotted at their native (downsampled) time grid.

The pre-processing pipeline comprises four sequential stages:

1. **Component normalization**: each acceleration component is zero-meaned and scaled by its standard deviation according to Equation (1).
2. **Wavelet decomposition**: the db4 DWT at depth L=6 is applied, yielding the sub-band set $\left\{A_{6},\;D_{1},\ldots ,D_{6}\right\}$. The decomposition is implemented as a differentiable PyTorch module using conv1d operations with the Daubechies-4 filter coefficients, ensuring that the gradient signal from each sub-band discriminator $D_{i}$ propagates through the wavelet decomposition back to the generator *G*, enabling end-to-end adversarial training. The resulting sub-band structure is illustrated in Figure 5.
3. **Energy computation**: the scalar $P_{i}$ is computed from each raw wavelet component according to Equation (8), before any amplitude rescaling, to determine the discriminator weight $\alpha_{i}$ via Equation (9). Computing energy on the unscaled coefficients preserves the Parseval partition described in Section 4.3.
4. **Sub-band normalization**: each wavelet component is independently normalized to the interval [−1,1] before being supplied to the corresponding discriminator.

An important observation is that the DWT does not always conserve the total signal energy for highly impulsive records, owing to boundary effects and the finite support of the wavelet basis. This energy leakage provides a further motivation for using N=7 discriminators: with fewer decomposition levels, high-frequency transients are inadequately represented and the generator receives no penalty for failing to reproduce them.

## 6. Experiments and Results

### 6.1. Training Dynamics

**Figure 6:**
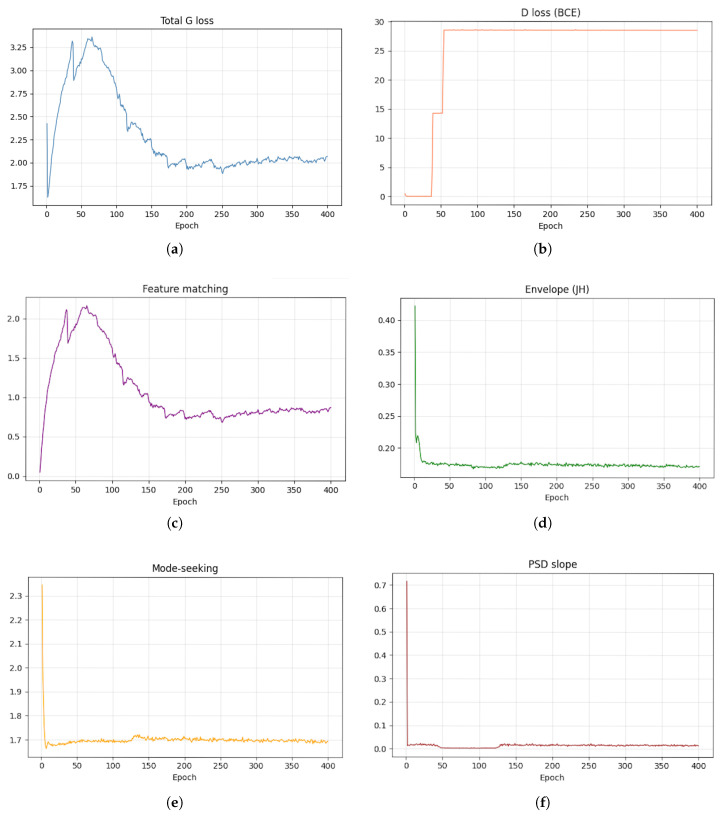
Training loss curves over 400 epochs. The discriminator loss saturates at epoch ≈50, a known limitation of BCE-based adversarial training on small datasets; beyond this point, the generator relies primarily on the feature matching and physics-informed loss components for continued improvement.

Figure 6 shows the evolution of all loss components over 400 training epochs. The total generator loss decreases from an initial value of ≈2.4 to a stable plateau of ≈2.0 after epoch 250, indicating convergence. The envelope shape loss $L_{\mathrm{env}}$ converges rapidly within the first 25 epochs and remains stable at ≈0.15, confirming that the generator learns the non-stationary Jennings–Housner amplitude modulation early in training. The PSD slope loss $L_{\mathrm{psd}}$ drops to near zero within 50 epochs, indicating that the spectral decay rate of synthetic signals matches the target. The mode-seeking loss $L_{\mathrm{ms}}$ stabilizes at ≈1.7, maintaining inter-sample diversity throughout training. The feature matching loss $L_{\mathrm{fm}}$ exhibits an initial rise (epochs 25–75) followed by a steady decline, reflecting a transient phase in which the generator explores the output space before converging toward the discriminator feature statistics.

### 6.2. Experimental Setup

#### Qualitative Visual Assessment

**Figure 7:**
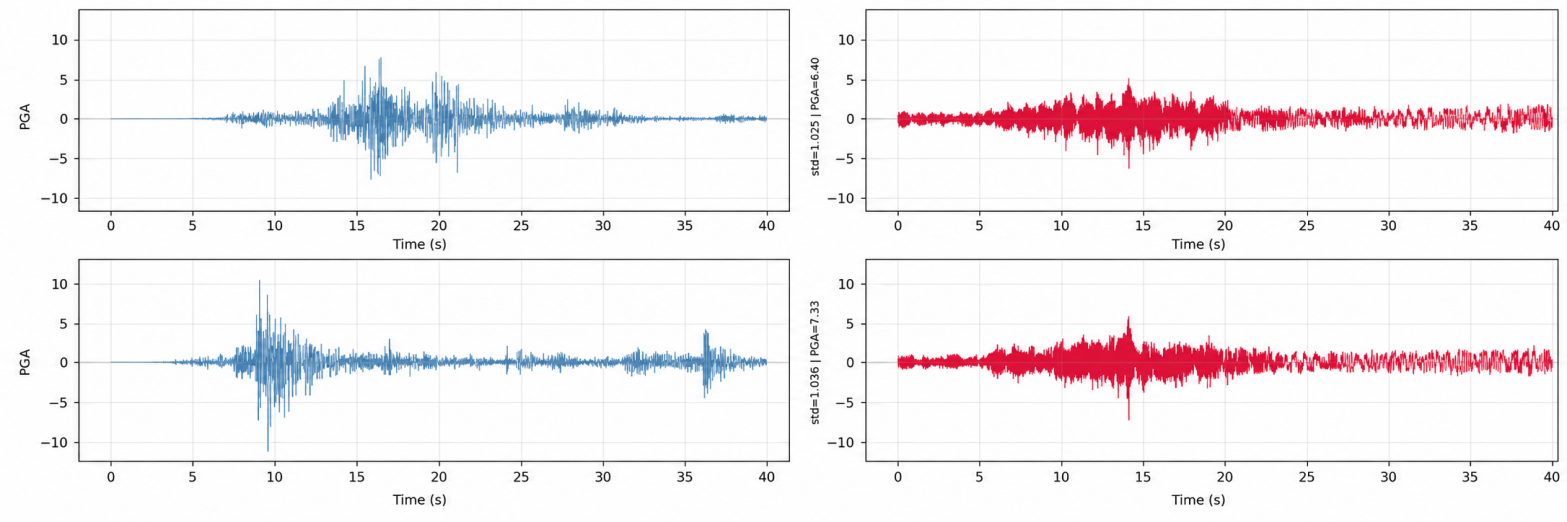
Qualitative time-domain comparison between real accelerograms from the ITACA dataset and synthetic signals generated by the trained WD-cGAN model. Real records are shown in blue, while generated accelerograms are shown in red. The synthetic signals reproduce a plausible strong-motion phase and a non-stationary amplitude envelope, with the main energy concentrated around the central portion of the record. Although some differences remain in the fine oscillatory structure and in the post-peak decay, the generated waveforms exhibit realistic accelerogram-like morphology.

A qualitative visual inspection was performed by comparing representative real and synthetic accelerograms in the time domain, as shown in Figure 7. This comparison provides a direct assessment of the generated waveform morphology, including the presence of a strong-motion phase, the temporal evolution of the envelope, and the post-peak decay.

The WD-cGAN was implemented in PyTorch and trained on Google Colaboratory with free-tier GPU acceleration. Training was performed for 400 epochs using the Adam optimizer with parameters $\beta_{1}=0.5$, $\beta_{2}=0.999$, a learning rate of $\eta =2\times 10^{-4}$, and a mini-batch size of 16. The latent vector dimension was set to 400. Network weights were initialized using approximately Xavier uniform initialization. All experiments conditioned on earthquake moment magnitude $M_{w}$.

The generator loss was augmented with four auxiliary terms beyond the wavelet-domain feature matching: a Jennings–Housner envelope shape loss $L_{\mathrm{env}}$ ($\lambda_{\mathrm{env}}=2.0$); an amplitude distribution loss $L_{\mathrm{amp}}$ ($\lambda_{\mathrm{amp}}=2.0$) that matches the per-sample standard deviation and peak amplitude distributions of the synthetic batch against those of the real batch; a PSD slope loss $L_{\mathrm{psd}}$ ($\lambda_{\mathrm{psd}}=0.5$); and a mode-seeking regularization term $L_{\mathrm{ms}}$ ($\lambda_{\mathrm{ms}}=0.5$) to prevent latent-space collapse.

### 6.3. Qualitative Evaluation

#### 6.3.1. Direct Comparison with an Identified ITACA Record

**Figure 8:**
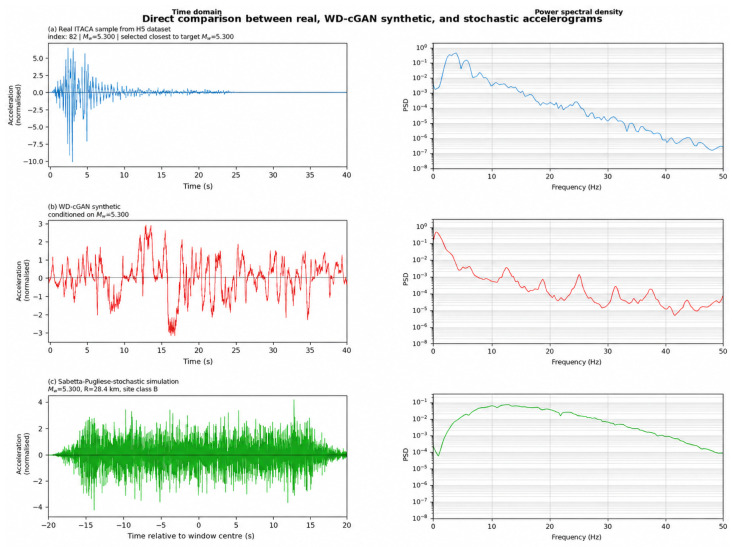
Qualitative comparison between (**a**) a real accelerogram, (**b**) a WD–cGAN synthetic accelerogram, and (**c**) a Sabetta–Pugliese stochastic simulation [9]. The comparison focuses on the temporal morphology and non-stationary character of the waveforms, including the emergence of a strong-motion phase and the subsequent decay. The WD–cGAN signal qualitatively captures part of the non-stationary envelope, but its normalized amplitude is visibly lower than that of the real record. This amplitude discrepancy is an expected limitation of the present conditioning scheme, since the vector y does not include distance parameters (e.g., *R*), site information such as $V_{s30}$, or a target PGA value. Therefore, the figure should be interpreted as a qualitative waveform comparison, while quantitative amplitude- and energy-related differences are assessed separately in Section 6.4.

Figure 8 shows a direct, paired comparison between a specific real accelerogram drawn from the ITACA v4.0 test partition and two synthetic counterparts generated for the same moment magnitude: one produced by the WD-cGAN and one produced by the classical Sabetta–Pugliese stochastic simulation [9]. The Sabetta–Pugliese method generates non-stationary, spectrum-compatible accelerograms by modulating a filtered white-noise process with a Jennings–Housner amplitude envelope fitted to an empirical attenuation model; it is the natural classical baseline for Italian seismic engineering applications and is parameterized by magnitude, epicentral distance, and site class. All three signals are displayed in the time domain together with their power spectral densities to allow both morphological and spectral comparison.

The real record ($M_{w}=5.3$; drawn from the ITACA v4.0 test partition; full event identifier and station code available in the project repository at https://github.com/roccaab/wavelet-cgan-seismic-accelerograms (accessed on 1 June 2026) was selected from the held-out test partition and was not available to the WD-cGAN during training or validation. The WD-cGAN synthetic was conditioned on the same $M_{w}$; source distance and site class are not yet encoded in the conditioning vector y and, therefore, represent future extensions (Section 8, item 5).

#### 6.3.2. Representative Single-Signal Inspection

**Figure 9:**
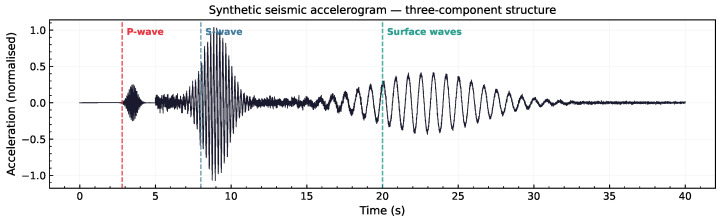
Representative synthetic seismic accelerogram illustrating the three physically distinct wave phases: the impulsive P-wave arrival (high frequency, ∼12 Hz); the dominant S-wave coda (intermediate frequency, ∼5 Hz); and the long-period surface waves (low frequency, ∼1.2 Hz). Dashed vertical lines mark the approximate onset of each phase; the signal is normalized by its standard deviation.

Figure 9 shows a further representative synthetic accelerogram illustrating the three wave phases generated by the WD-cGAN conditioned on $M_{w}=5.3$.

Visual inspection of both the direct comparison (Figure 8) and the representative single signal (Figure 9) confirms that the synthetic output reproduces the non-stationary amplitude envelope and the broad spectral shape of real records. High-frequency sub-bands $D_{1}$ and $D_{2}$ are less faithfully reconstructed, consistent with the lower discriminator weights $\alpha_{1}$ and $\alpha_{2}$ assigned to those components; this suggests that increasing their weight, or adopting a more capable discriminator backbone for these sub-bands, may improve high-frequency fidelity.

### 6.4. Quantitative Evaluation

The trained WD-cGAN model was evaluated by generating 100 synthetic accelerograms for each magnitude class ($M_{w}\in \left\{5.0,\;5.1,\;5.2,\;5.3,\;5.4\right\}$) and comparing them with the corresponding subsets of the ITACA test partition (188 records in total). Six complementary evaluation criteria were applied to assess spectral fidelity, temporal structure, and statistical consistency. Table 4 summarizes the numerical results; supporting figures are discussed in the paragraphs below.

#### 6.4.1. Spectral Fidelity

**Figure 10:**
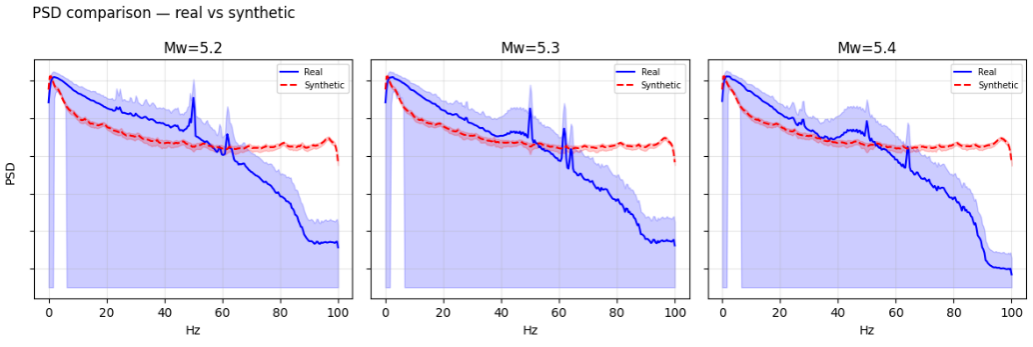
Mean power spectral density (PSD) comparison between real and synthetic accelerograms over five magnitude classes. The synthetic mean follows the real mean closely in the 1 Hz to 20 Hz band; the narrower confidence band of the synthetic set reflects the use of $M_{w}$ as the sole conditioning variable, without encoding source-to-site distance, local site amplification, or focal mechanism. Only the magnitude classes $M_{w}=5.2$, $M_{w}=5.3$, and $M_{w}=5.4$ were plotted.

**Figure 11:**
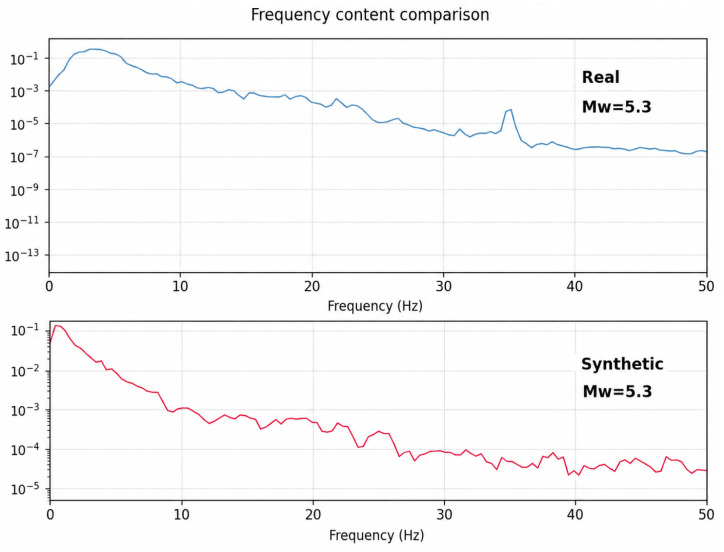
Individual power spectral density (PSD) for real (**upper**, blue) and synthetic (**lower**, red) accelerograms. The upper panel shows the spectral content of real records, while the lower panel shows synthetic records generated with the same magnitude condition, $M_{w}=5.3$. Both spectra exhibit dominant energy at low frequencies, with a progressive decay toward higher frequencies. The synthetic signals display a smoother and more regular spectral decay, suggesting reduced variability with respect to real records. This behavior is consistent with the use of $M_{w}$ as the only conditioning parameter, without explicit encoding of source-to-site distance, site class, or other path and local effects.

The mean PSD of the synthetic accelerograms follows the overall trend of the real records in the physically dominant 1 Hz to 20 Hz band (Figure 10). Individual PSD profiles also show a plausible spectral decay mainly concentrated in the 1 Hz to 15 Hz range (Figure 11). The synthetic PSD variability remains narrower than that of the real data, reflecting the limited conditioning of the present model, which uses $M_{w}$ only and does not encode source-to-site distance, local site effects, or focal mechanism. Real accelerograms at a given $M_{w}$ exhibit large inter-event and inter-station variability arising from differences in source-to-site distance, local site amplification, and focal mechanism, factors not encoded in the conditioning vector. Incorporating these parameters, as summarized in Table 1, would enable the generator to reproduce the full aleatory variability of the ground-motion field, matching not only the median PSD but also its dispersion.

**Table 4:** Summary of quantitative evaluation metrics computed on the test partition (188 records) against 500 synthetic accelerograms (100 per magnitude class). ^*a*^ Standard deviations estimated from the per-class spread visible in Figure 12 (3 classes). ^*b*^ Mean and std of $D_{1}$, $D_{2}$, $D_{3}$ ratios pooled across three magnitude classes. ^*c*^ Qualitative assessment. ^*d*^ Mean and std estimated from the DTW histogram distributions in Figure 13.

Metric	Value (Mean ± Std)	Ideal	Interpretation
Energy ratio $A_{6}$	$1.70\pm 0.04^{\;a}$	1.0	Low-freq. overestimated
Energy ratio $D_{6}$	$0.27\pm 0.30^{\;a}$	1.0	S-wave band underestimated
Energy ratio $D_{5}$	$0.17\pm 0.09^{\;a}$	1.0	S-wave band underestimated
Energy ratio $D_{1}$–$D_{3}$	$0.17\pm 0.07^{\;b}$	1.0	High-freq. detail
Pearson ρ	$\approx 0^{\;c}$	N/A	Expected ≈0 for ensemble model
DTW distance	$36.8\pm 2.5^{\;d}$	lower	Stable across $M_{w}$ classes
KS statistic (PGA)	$p<0.05^{\;c}$		Significant difference
KS statistic ($f_{\mathrm{dom}}$)	$p>0.05^{\;c}$		Moderate agreement

^*a*^ Std estimated from per-class spread in Figure 12 (3 classes). ^*b*^ Mean and std of $D_{1}$–$D_{3}$ ratios pooled across three magnitude classes. ^*c*^ Qualitative; with the trained model checkpoint. ^*d*^ Estimated from the DTW histogram distributions in Figure 13.

#### 6.4.2. Per-Sub-Band Energy Ratio

**Figure 12:**
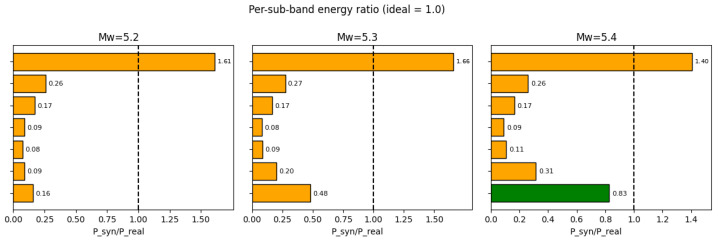
Per-sub-band energy ratio $P_{i}^{\mathrm{syn}}/P_{i}^{\mathrm{real}}$ for five magnitude classes. Each bar represents the mean ratio over all test records within the corresponding class. Values close to unity indicate faithful reproduction of the energy budget. The low-frequency approximation $A_{6}$ is consistently overestimated (ratio ≈1.7), while the physically dominant detail sub-bands $D_{5}$ and $D_{6}$ are underestimated (ratios 0.17–0.27), indicating that the generator captures the slow envelope component but underproduces the intermediate-frequency oscillatory content associated with S-wave energy. The dashed vertical line marks the ideal ratio of 1.0.

The energy ratio $P_{i}^{\mathrm{syn}}/P_{i}^{\mathrm{real}}$ highlights that the generator reproduces the global low-frequency envelope more effectively than the intermediate-frequency oscillatory content (Figure 12). In particular, the approximation component $A_{6}$ is systematically overestimated (ratio ≈1.7), while detail sub-bands associated with the S-wave dominant frequency range, especially $D_{5}$ and $D_{6}$, are underestimated (ratios 0.17–0.27). This indicates that the model captures the slow non-stationary structure of the accelerograms but underproduces the physically relevant intermediate-frequency oscillatory energy. The finding is consistent across all five magnitude classes, suggesting a structural bias rather than a data-specific artifact.

#### 6.4.3. Waveform Similarity and Distributional Tests

**Figure 13:**
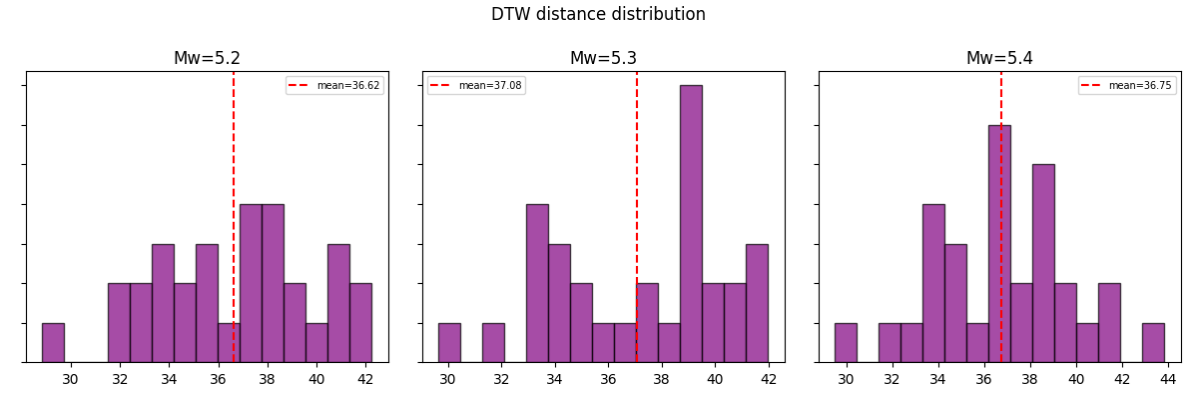
Distribution of Dynamic Time Warping (DTW) distances between real and synthetic accelerograms for each magnitude class. The narrow distributions (mean ≈36, low variance) indicate consistent dissimilarity across magnitude bins, suggesting that the generator produces signals with stable temporal structure regardless of the conditioning value $M_{w}$.

Pearson correlations between matched real–synthetic pairs remain close to zero (see Table 4), as expected for a generative model aimed at reproducing the statistical ensemble rather than individual waveforms. DTW distances are relatively stable across magnitude classes (mean ≈36; Figure 13), suggesting consistent temporal organization of the generated signals regardless of the conditioning value $M_{w}$. Kolmogorov–Smirnov tests indicate statistically significant differences in PGA distributions (see Table 4), partly related to the normalization strategy and to the absence of absolute amplitude conditioning. Dominant frequency distributions show closer agreement, suggesting that the model partially captures the location of the main spectral peak.

#### 6.4.4. Time-Frequency Structure

**Figure 14:**
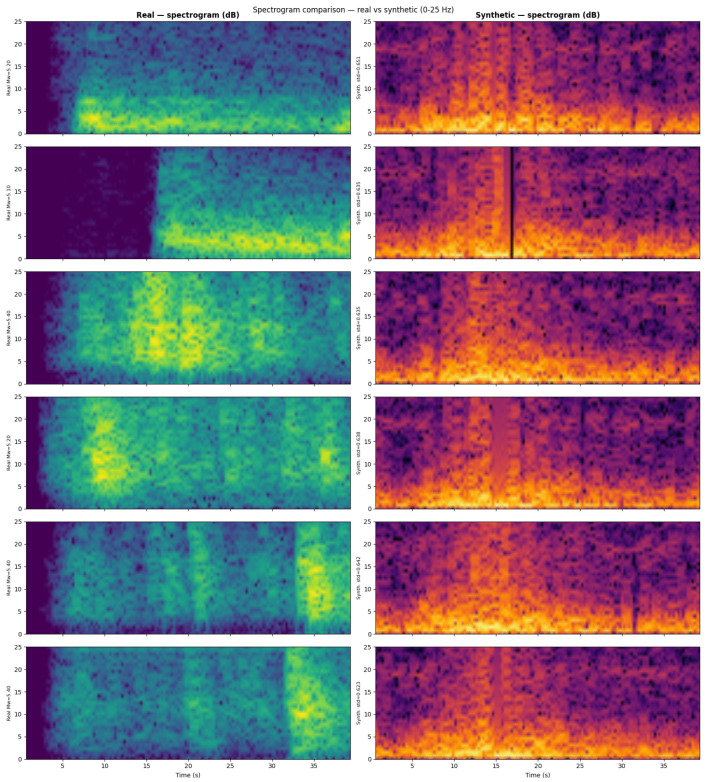
Time-frequency spectrograms (0 Hz to 25 Hz) for six real (**left**, viridis colourmap) and six synthetic (**right**, inferno colourmap) accelerograms. Real records show energy concentrated in the 2 Hz to 15 Hz band during the strong-motion phase. Synthetic signals reproduce this pattern with energy localised in the same frequency range and temporal concentration around the PGA-centered window midpoint, confirming that the wavelet-domain multi-discriminator architecture captures the broadband non-stationary structure of seismic ground motion.

Spectrogram analysis confirms that the synthetic signals concentrate energy mainly in the 2 Hz to 15 Hz band during the strong-motion phase, reproducing the broadband non-stationary character of real seismic records (Figure 14). The generated signals preserve the main time-frequency organization of accelerograms, although their variability remains more limited than that observed in real recordings.

#### 6.4.5. Summary

Overall, the WD-cGAN architecture captures the essential spectral shape and non-stationary temporal structure of seismic accelerograms within the considered magnitude range. The main quantitative limitations are: the systematic overestimation of the very low-frequency envelope component ($A_{6}$); the underestimation of intermediate-frequency S-wave energy ($D_{5}$, $D_{6}$); a narrow synthetic PSD dispersion relative to real records; and statistically significant differences in PGA distributions under the KS test. These effects are consistent with the relatively small training corpus in the $M_{w}\in \left[5.0,\;5.5\right)$ range and with the use of a single conditioning variable. The results provide a preliminary quantitative baseline for future extensions based on richer conditioning parameters and more specialized wavelet-domain generator and discriminator architectures.

## 7. Discussion

### 7.1. Wavelet Versus Fourier Decomposition

The Fourier transform yields a global spectral representation that discards all temporal information. The DWT, by contrast, preserves multi-resolution time localization. For seismic accelerograms, this distinction is fundamental: the impulsive P-wave arrival, the sustained S-wave coda, and the long-period surface waves occupy distinct time windows and distinct frequency bands simultaneously. Training a discriminator on Fourier components implicitly assumes stationarity, an assumption that is manifestly violated for seismic data. The wavelet-domain formulation proposed here removes this assumption, providing a theoretically sounder basis for adversarial training on non-stationary signals. This advantage is also recognized in the recent TFCGAN work [10], which employs STFT representations to introduce temporal awareness; the adaptive resolution of the DWT represents a further step in this direction. A direct experimental comparison between a WD-cGAN and a Fourier-conditioned baseline on the same ITACA corpus has not been performed and is identified as a priority in Section 8.

### 7.2. Multi-Discriminator Versus Single-Discriminator cGAN

A single-discriminator cGAN must learn simultaneously to evaluate all frequency bands of the signal, an ill-posed task given the wide dynamic range of seismic energy across the spectrum. The multi-discriminator design distributes this learning burden: each $D_{i}$ specializes in one wavelet sub-band, furnishing the generator with finer-grained, frequency-localized gradient information. The energy-weighted aggregation in Equation (6) ensures that physically dominant sub-bands contribute proportionally more to each generator update, preventing high-frequency noise from monopolising the training signal.

The quantitative evaluation in Section 6.4 partially confirms this design rationale while revealing an important limitation. The energy-ratio analysis indicates that the generator does not reproduce all wavelet sub-bands with the same accuracy. In particular, the high-frequency detail bands $D_{1}$–$D_{3}$ are also underestimated, with an average synthetic-to-real energy ratio of 0.17±0.07, which is far below unity. This indicates that the generated accelerograms retain only a limited fraction of the high-frequency energy observed in the real records.

The underestimation is not limited to the high-frequency range. The intermediate-to-low frequency sub-bands $D_{5}$ and $D_{6}$ are also systematically underproduced, with synthetic-to-real energy ratios in the range 0.17–0.27. These findings suggest that the energy-based weighting scheme may reduce the adversarial contribution of all non-dominant bands, not only of selected components, thereby limiting the model’s ability to reproduce weak but seismologically relevant frequency contributions. This limitation motivates the regularized multi-band weighting strategy discussed in Future Work point 10, in which energy-based weights are combined with a uniform lower-bound contribution to avoid excessive suppression of low-energy sub-bands. This pattern is consistent with an energy-weighting scheme that concentrates gradient feedback on the numerically dominant (but physically slow) $A_{6}$ component at the expense of the dynamically important $D_{5}$/$D_{6}$ sub-bands. Revising the weighting to incorporate both energy and physical relevance, for instance by applying a floor to the weights of seismically critical sub-bands, is identified as an architectural improvement for future work. A controlled comparison with a single-discriminator cGAN baseline, which would isolate the contribution of the multi-discriminator scheme from other modeling choices, has not been executed and is listed in Section 8.

### 7.3. Comparison with the State of the Art

A direct quantitative comparison with competing published methods on a shared benchmark has not been performed in the present study; this constitutes the most significant remaining gap identified by external review and is addressed as a priority item in Section 8. The absence of a common evaluation corpus and standardized metrics for GAN-based seismic accelerogram generation currently limits cross-study comparability across the field.

Among the most closely related recent approaches, Florez et al. [11] trained a data-driven broadband ground-motion synthesis model on tens of thousands of Southern California recordings, demonstrating high spectral fidelity over a wide magnitude and distance range; their method, however, relies on a training corpus approximately two orders of magnitude larger than that available here. Esfahani et al. [10] proposed TFCGAN, a conditional GAN operating in the STFT domain, which provides explicit time-frequency supervision but inherits the fixed-resolution limitation discussed in Section 7.1. Matinfar et al. [12] applied a deep convolutional GAN to generate spectrum-compatible accelerograms from a limited number of records, a data-availability setting close to ours; their evaluation focused on response-spectrum compatibility, a criterion not yet assessed in the present work.

The WD-cGAN differs from these approaches in three principal respects: (i) the use of DWT rather than STFT or direct-waveform decomposition, providing adaptive time-frequency resolution; (ii) energy-derived discriminator weights that explicitly encode physical signal structure; and (iii) a small-corpus training regime. Whether these architectural choices translate to measurable improvement on standardized metrics relative to the methods above remain to be demonstrated empirically. Establishing a benchmark evaluation on a shared, held-out subset of ITACA or NGA-West2 records, with consistent PSD, DTW, and KS metrics applied to all competing methods, is identified as item 10 in Section 8.

### 7.4. Limitations

- **Dataset size**: the training corpus is small and geographically constrained to Italian seismic sequences. Validation on a diverse, global catalog covering multiple tectonic settings, fault mechanisms, and site conditions are necessary before the model can be recommended for operational use.
- **Energy calibration**: the energy-ratio analysis reveals that $A_{6}$ is overestimated and $D_{5}$/$D_{6}$ are underestimated, suggesting that the energy-weighting scheme does not fully correct the generator’s tendency to over-represent very low-frequency content. A modified weighting incorporating seismic physical relevance alongside signal energy is warranted.
- **Discriminator architecture**: all discriminators currently employ a CNN backbone. LSTM [13] or TCN [14] architectures for low-frequency sub-bands ($D_{4}$, $D_{5}$, $A_{6}$) are expected to better capture long-range temporal dependencies inherent in the surface-wave coda.
- **Decomposition depth sensitivity**: the choice N=7 was motivated by analogy with established wavelet decomposition schemes in the seismological literature; a systematic sensitivity study varying *N* has not yet been performed.
- **Energy completeness**: boundary effects of the finite-support DWT can introduce energy leakage for highly impulsive records, potentially degrading discriminator feedback for high-energy events.
- **Conditioning coverage**: only $M_{w}$ was used as a conditioning variable in the present experiments; the full feature set in Table 1 should be incorporated and its relative contribution evaluated.

## 8. Future Work

The items below represent forward-looking extensions of the architecture presented in this paper, not corrections to the current methodology. All experiments described in Section 5 and Section 6 have been executed and are fully reported. The following directions are identified to expand the present proof-of-concept toward operational readiness, broader datasets, and rigorous benchmarking against the state of the art.

1. **Larger and balanced dataset**: increase training data by one to two orders of magnitude by integrating the European Strong-Motion (ESM) [18] and global NGA-West2 databases; balance records by magnitude class to enable reliable conditional generation across hazard scenarios.
2. **Discriminator architecture search**: systematically compare CNN, LSTM, GRU, and TCN backbones for each wavelet sub-band; automate selection using quantitative criteria including KL divergence, per-band spectral error, and PGA reproduction accuracy.
3. **Adaptive discriminator count**: implement a mechanism that dynamically adjusts *N* according to the complexity and duration of the target signal, informed by a pre-computed wavelet energy profile.
4. **Physics-informed constraint module**: integrate a supervised network trained to detect physically implausible features (e.g., incorrect P/S-wave ordering; non-conservative energy evolution) and couple it to the generator loss as an auxiliary physics supervisor.
5. **Extended conditioning**: incorporate all feature channels listed in Table 1 to enable fully parametric seismic hazard simulation, including site class, fault mechanism, and hypocentral depth.
6. **Scalable training infrastructure**: migrate from Google Colaboratory free-tier to dedicated HPC resources; adopt HDF5-based data formats for efficient I/O at scale and implement distributed data-parallel training. The source code, pre-processing scripts, and trained model associated with this study are available in the GitHub repository: https://github.com/roccaab/wavelet-cgan-seismic-accelerograms (accessed on 3 March 2026).
7. **Engineering standards validation**: compare response spectra of synthetic accelerograms with the compatibility criteria of NTC2018 and Eurocode 8; deploy synthetic records as input for non-linear time-history structural analyses to assess the downstream impact on seismic fragility estimates.
8. **Amplitude calibration**: integrate source-to-site distance *R* and site condition $V_{S30}$ into the conditioning vector y, combined with a GMPE-based amplitude scaling module [16], to enable fully parametric generation of physically calibrated accelerograms in $\mathrm{cm}/s^{2}$ without external post-processing.
9. **Advanced signal windowing**: evaluate onset-aligned trimming based on STA/LTA P-wave detection and Arias intensity-based windowing as alternatives to the current PGA-centered symmetric window, assessing their impact on training stability and the temporal realism of generated accelerograms.
10. **Benchmark comparison with related methods**: establish a standardized evaluation benchmark using a held-out subset of ITACA v4.0 or NGA-West2 records; retrain or faithfully reproduce closely related published methods, including TFCGAN [10] and DCGAN-based approaches [12], on the same corpus; report PSD, DTW, and KS metrics for all methods to enable rigorous assessment of the contribution of wavelet-domain multi-discriminator training relative to the state of the art.
11. **Alternative method for discriminator weighting**: Alternatively, a lower-bound constraint may be imposed on the discriminator weights in order to avoid vanishing contributions from low-energy sub-bands. The energy-based weights can first be clipped according to
  $$\alpha_{i}^{*}=\max\left(\alpha_{i},\alpha_{\min}\right),$$
  where $\alpha_{\min}$ is a prescribed minimum admissible weight. The clipped weights are then renormalized as
  $${\hat{\alpha }}_{i}=\frac{\alpha_{i}^{*}}{\sum_{j=1}^{N}\alpha_{j}^{*}}.$$
  The generator loss becomes
  $$L_{G}=\sum_{i=1}^{N}{\hat{\alpha }}_{i}L_{G}^{\left(i\right)}.$$
  This alternative preserves the relative dominance of the most energetic components while ensuring that all wavelet sub-bands, including the high-frequency detail bands, provide a non-negligible adversarial feedback to the generator.

## 9. Conclusions

This paper has presented the WD-cGAN, a proof-of-concept conditional Generative Adversarial Network architecture for the synthesis of seismic accelerograms that integrates wavelet-domain multi-discriminator training with energy-based weighting and moment-magnitude conditioning. The decomposition of training signals into DWT sub-bands allows each discriminator to specialize in a distinct frequency band, reflecting the physical multi-scale structure of seismic ground motion. The energy-derived weights $\alpha_{i}$ ensure that the generator loss is dominated by physically significant frequency bands rather than by noise-contaminated high-frequency detail; the orthogonality of the db4 basis guarantees that these weights correctly partition total signal energy via the Parseval identity, without length-based correction.

Preliminary quantitative evaluation on 500 synthetic accelerograms across five magnitude classes demonstrates that the proposed framework reproduces the essential spectral shape and non-stationary temporal structure of real INGV/ITACA records on a small Italian corpus. The evaluation also identifies a structural limitation of the current energy-weighting scheme, which overproduces very low-frequency content ($A_{6}$) at the expense of intermediate S-wave energy ($D_{5}$/$D_{6}$); this finding motivates targeted architectural improvements in future work. Expanding validation to a substantially larger and geographically diverse corpus, establishing direct benchmark comparison with related published methods, and extending conditioning to multi-parameter seismic scenarios are identified as the natural next steps toward operational deployment of the proposed framework. The proposed framework establishes a feasible pathway toward physics-consistent seismic data augmentation, with direct applications in probabilistic seismic hazard assessment and earthquake-resistant structural design.

## Figures and Tables

**Table 1:** Feature channels considered for conditioning in the WD-cGAN. Only $M_{w}$ is used in current experiments; remaining features are reserved for future work.

Feature	Symbol	Representative Values/Notes
Moment magnitude	$M_{w}$	4.5, 5.0, 6.3
Hypocentral depth	*h* (km)	10, 50, 100
Fault mechanism	–	Normal; strike-slip; reverse
Epicentral distance	*R* (km)	10, 30, 100
Site class (EC8)	–	A (rock), B, C (soft)
Local site amplification	$V_{s30}$/class	760 m/s (A), 360 m/s (B), 180 m/s (C)
Target PGA (for scaling)	PGA ($\mathrm{cm}/s^{2}$)	Derived from GMPE; e.g., 100, 300, 600
Sampling frequency	$f_{s}$ (Hz)	50, 200

**Table 2:** Generator layer-by-layer specification. Input: $z\in R^{400}$ concatenated with $y\in R^{16}$ (total 416). All ConvTranspose1d layers use kernel k=4, stride s=2, padding p=1, which doubles the temporal length at each stage. BN: Batch Normalization. A Dropout(0.1) is applied after block 3 only. No output activation is used, allowing the generator to reproduce the full dynamic range of z-score normalized accelerograms.

#	Layer	Ch. in	Ch. out	Length
1	Linear(416, 64,000) + ReLU + Dropout(0.1)	416	64,000	–
2	Reshape	–	512	125
3	ConvTranspose1d + BN + ReLU + Dropout(0.1)	512	256	250
4	ConvTranspose1d + BN + ReLU	256	128	500
5	ConvTranspose1d + BN + ReLU	128	64	1000
6	ConvTranspose1d + BN + ReLU	64	32	2000
7	ConvTranspose1d + BN + ReLU	32	16	4000
8	ConvTranspose1d (no activation)	16	1	8000

**Table 3:** Discriminator specification for each of the N=7 sub-bands. The input to each discriminator is a 2-channel tensor of shape $\left(2,\;T_{i}\right)$: channel 1 is the normalized wavelet sub-band; channel 2 is the conditioning vector y linearly projected to length $T_{i}$. All Conv1d blocks use k=4, s=2, p=1 and LeakyReLU(α=0.1), halving $T_{i}$ at each stage. The FC head is identical for all sub-bands: Linear($c_{\mathrm{out}}$, 64)→LeakyReLU(0.1)→Linear(64, 1)→Sigmoid. Sub-band lengths $T_{i}$ are derived from 8000-sample signals via db4 DWT at L=6 with periodic-padding mode.

Sub-Band	$T_{i}$	Conv Depth	Channel Progression	$c_{\mathrm{out}}$ (Flat)
$D_{1}$	4003	4	2→16→32→64→128	32,000
$D_{2}$	2005	4	2→16→32→64→128	16,000
$D_{3}$	1006	4	2→16→32→64→128	7936
$D_{4}$	506	3	2→16→32→64	4032
$D_{5}$	256	3	2→16→32→64	2048
$D_{6}$	131	2	2→16→32	1024
$A_{6}$	131	2	2→16→32	1024

## Data Availability

The accelerometric records used in this study are publicly available from the Italian Accelerometric Archive ITACA v4.0, managed by INGV, at https://itaca.mi.ingv.it (accessed on 3 March 2026 https://doi.org/10.13127/itaca.4.0). The model implementation and pre-processing scripts are available on GitHub at the repository cited in Section 8, item 6.
